# Ferroptosis: Emerging mechanisms, biological function, and therapeutic potential in cancer and inflammation

**DOI:** 10.1038/s41420-024-01825-7

**Published:** 2024-01-24

**Authors:** Xin Jin, Jiuren Tang, Xiangyu Qiu, Xiaoya Nie, Shengming Ou, Geyan Wu, Rongxin Zhang, Jinrong Zhu

**Affiliations:** 1 School of Life Sciences and Biopharmaceutics, Guangdong Pharmaceutical University, Guangzhou, China; 2 Biomedicine Research Centre, Guangdong Provincial Key Laboratory of Major Obstetric Diseases, Guangdong Provincial Clinical Research Center for Obstetrics and Gynecology, The Third Affiliated Hospital of Guangzhou Medical University, Guangzhou Medical University, Guangzhou, China; 3 State Key Laboratory of Oncology in South China, Collaborative Innovation Center for Cancer Medicine, Sun Yat-sen University Cancer Center, Guangzhou, China

**Keywords:** Cancer therapy, Cancer

## Abstract

Ferroptosis represents a distinct form of programmed cell death triggered by excessive iron accumulation and lipid peroxidation-induced damage. This mode of cell death differentiates from classical programmed cell death in terms of morphology and biochemistry. Ferroptosis stands out for its exceptional biological characteristics and has garnered extensive research and conversations as a form of programmed cell death. Its dysfunctional activation is closely linked to the onset of diseases, particularly inflammation and cancer, making ferroptosis a promising avenue for combating these conditions. As such, exploring ferroptosis may offer innovative approaches to treating cancer and inflammatory diseases. Our review provides insights into the relevant regulatory mechanisms of ferroptosis, examining the impact of ferroptosis-related factors from both physiological and pathological perspectives. Describing the crosstalk between ferroptosis and tumor- and inflammation-associated signaling pathways and the potential of ferroptosis inducers in overcoming drug-resistant cancers are discussed, aiming to inform further novel therapeutic directions for ferroptosis in relation to inflammatory and cancer diseases.

## Facts

- Ferroptosis represents a distinct iron-dependent form of cell death, and the process of cell death is usually accompanied by iron accumulation and lipid peroxidation.
- Ferroptosis is involved in the development of a variety of diseases, such as neurological disorders, tissue ischemia-reperfusion injury, and cancer.
- Ferroptosis has a dual effect on promoting and suppressing tumors in experimental tumor models.
- Ferroptosis inducer therapy is a promising new anticancer therapy.

## Open questions

- Inflammation has an impact on ferroptosis, but what precisely is the underlying mechanism?
- Whether definitive biomarkers of ferroptosis can be identified in the future could prove invaluable in studying the biological function of ferroptosis.
- Ferroptosis inducers may offer new therapeutic opportunities for many diseases, but their specific applications need to be further investigated.

## Introduction

Ferroptosis, as proposed by Dixon in 2012, is an iron-dependent form of regulated cell death (RCD) that exhibits distinct morphological and biochemical features compared to other cell death forms [1]. The critical characteristics of ferroptosis are marked by iron accumulation and lipid peroxidation. Consequently, intracellular iron accumulation and lipid peroxidation are the primary drivers of ferroptosis occurrence [1]. Current cancer treatment strategies aim to selectively eliminate cancer cells without causing harm to healthy cells [2]. Traditional antitumor drugs are successful in killing tumor cells by triggering apoptosis. However, the ability of apoptosis to remove tumor cells is restricted, and these traditional therapies are susceptible to drug resistance [3]. Therefore, alternative methods of inducing non-apoptosis present new possibilities for treating tumors. Apoptosis is one of the most extensively researched regulated cell death forms [4]. As a unique RCD, ferroptosis has attracted much attention. Aberrant activation of ferroptosis pathways is a hallmark of various pathologies, such as malignancies and chronic inflammatory disorders. Recently, significant advancements have been achieved in exploring ferroptosis in cancer cells [3]. In addition, cancer-related signaling pathways can regulate ferroptosis in cancerous cells. Cancer cells display an exceptional neoplastic metabolism and heightened reactive oxygen species (ROS) attributes [5–8]. Thus, specific cancer cells are more vulnerable to ferroptosis. Moreover, various tumor suppressors, such as p53 and BRCA1-associated protein 1 (BAP1), have been discovered to deter cancer progression by obstructing ferroptosis [9]. Therefore, targeting ferroptosis may provide new therapeutic opportunities to treat cancers refractory to conventional therapies. On the other hand, ferroptosis, a newly discovered form of cell death, may contribute to inflammation and activate intrinsic immunity [10]. Studies have shown that ferroptosis is linked to elevated prostaglandin-endoperoxide synthase 2 (PTGS2) expression and prostaglandin E2 (PGE2) secretion, which accelerates arachidonic acid metabolism, releasing inflammatory mediators and activating the inflammatory response [11]. Ferroptosis may also involve the inflammatory process by producing lipoxygenase (LOX) and cyclooxygenase (COX) products [12]. This review aims to link cancer and ferroptosis, determine the influence of molecular mechanisms associated with the ferroptosis pathway on the occurrence and development of inflammation and cancer, and provide new ideas and effective targets for the prevention and treatment of cancer.

## Characteristics associated with ferroptosis

### Morphological features

One study suggests that ferroptosis is morphologically and biochemically distinct from autophagy, apoptosis, and necrosis [1]. It differs from the morphological characteristics of typical necrosis and does not exhibit features such as rupture of the plasma membrane, cytoplasmic swelling, and moderate chromatin condensation. Unlike traditional apoptosis, apoptosis is characterized by plasma membrane blebbing, pseudopod retraction, reduction of cellular and nuclear volume, formation of apoptotic bodies and nuclear fragmentation, and chromatin condensation. Ferroptosis does not possess autophagic characteristics, for example, the accumulation of autophagic vacuoles (enclosed double-layer membrane structures). Ferroptosis is mainly characterized by small mitochondria, outer mitochondrial rupture, intact plasma membrane, normal nuclear size, and absence of chromatin condensation [13–17] (Table 1).

**Table 1:** The features of ferroptosis, apoptosis, autophagy, Necroptosis, and Pyroptosis [13–17].

Cell death	Ferroptosis	Apoptosis	Autophagy	Necroptosis	Pyroptosis
Morphological features	Small mitochondria, outer mitochondrial rupture, no rupture of the plasma membrane, normal nuclear size, and no chromatin condensation	Plasma membrane blebbing, pseudopod retraction, reduction of cellular and nuclear volume, formation of apoptotic bodies and nuclear fragmentation, and chromatin condensation	Formation of double-membraned autolysosomes	Plasma membrane rupture, swelling of the cytoplasm and organelles, moderate chromatin condensation	Karyopyknosis, rupture of the cell edema and membrane
Biochemical features	Iron accumulation and lipid peroxidation	DNA fragmentation	Increased lysosomal activity	Drop in ATP levels, activation of RIP1, RIP3, and MLKL	Dependent on caspase-1 and proinflammatory cytokine releases
Regulatory pathways	System Xc- and GPX4, p62-Keap-NRF2 pathway, P53/SLC7A11, P53-SAT1-ALOX15 pathay, p53-DPP4-NOX1 pathway, p53-CDKN1A/P21 pathway	Death receptor, Mitochondrial pathway, and Endoplasmic reticulum pathway, Caspase, p53, Bcl-2 mediated signaling pathway	PI3K-AKT-mTOR, MAPK-ERK1/2-mTOR signal pathway, Beclin-1, p53 signaling pathway	TNFα, TNFR1, TLR3, TRAIL, Fasl and ROS related signaling pathways; PKC-MAPK-AP-1 mediated signaling pathway	Caspase-1, NLRP3-related signaling pathways
Key genes	GPX4, NRF2, TRF1, SLC7A11, P53	Caspase, p53, Fas, Bcl-2, Bax	ATG, ATG7, LC3, Beclin-1, DRAM3, TFEB	RIP1, RIP3, LEF1	Caepase-2, IL-1β, IL-18

### Biochemical features

Biochemical features in ferroptosis involve iron accumulation, lipid peroxidation, generation of ROS, and depletion of glutathione peroxidase 4 (GPX4). Among these, the crucial factors are iron accumulation and lipid peroxidation [1, 13] (Table 1).

### Iron accumulation

Iron is a metal with redox properties and is also one of the essential trace elements in the body. The abnormal distribution and content of iron in the body will impact normal physiological activities [3, 18]. The activation of ferroptosis, facilitated by agents such as Erastin and RSL3, occurs through the accumulation of iron within cells. This process not only directly generates excessive ROS by initiating the non-enzymatic Fenton reaction but also enhances the activity of lipoxygenase (ALOX) or EGLN prolyl hydroxylases (also known as PHD) [1, 19, 20].

### Lipid peroxidation

Lipid peroxidation is one of the key signals that initiate membrane oxidative damage during ferroptosis [1]. Lipid peroxidation can divide into non-enzymatic lipid peroxidation (auto-oxidation) and enzymatic lipid peroxidation, which is a reaction catalyzed by free radicals. ROS in auto-oxidation initiates the oxidation of polyunsaturated fatty acids (PUFAs), especially arachidonic acid peroxide and adrenalin, leading to the accumulation of peroxides. Enzymatic lipid peroxidation is regulated by LOX activity [3]. It can catalyze the production of a variety of lipid hydroperoxides from PUFAs. Fatty acids include saturated fatty acids (no double bonds), monounsaturated fatty acids (MUFAs, one double bond), and polyunsaturated fatty acids (PUFAs, >1 double bond) [21]. PUFAs (including linoleic acid and arachidonic acid) can stimulate RSL3-induced ferroptosis, while oleic acid, a monounsaturated fatty acid (MUFA), may protect cells from ferroptosis through neutralization [19]. Thus, it has an inhibitory effect on ferroptosis.

## Regulation of ferroptosis

### Iron metabolism

**Fig. 1:**
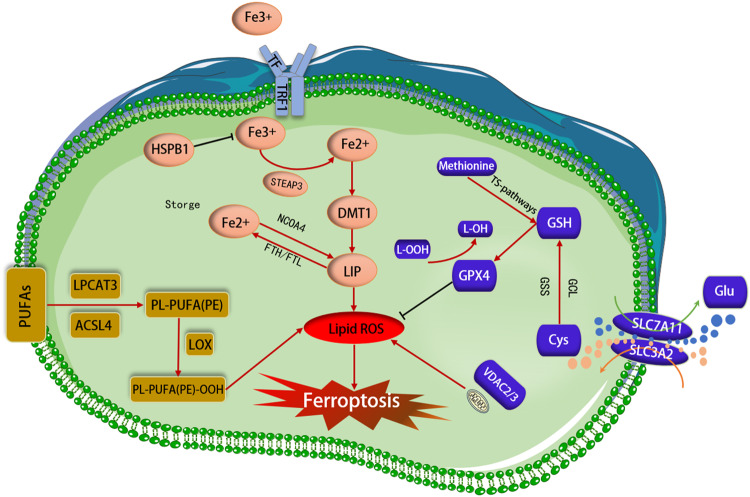
Mechanisms of ferroptosis. The figure shows the primary metabolic pathways regulating ferroptosis: iron metabolism, lipid metabolism, and amino acid metabolism. ACSL4 acyl-CoA synthetase long-chain family member 4, DMT1 divalent metal transporter 1, GPX4 glutathione peroxidase 4, GSH glutathione, GSS glutathione synthetase, GCL glutamate-cysteine ligase, HSPB1 heat shock protein beta-1, IREB2 iron response element-binding protein 2, LOX lipoxygenase, LPCAT3 lysophosphatidylcholine acyltransferase 3, NCOA4 nuclear receptor coactivator 4, FTL ferritin light chain, FTH1 ferritin heavy chain 1, NRF2 nuclear factor E2-related factor 2, PUFA polyunsaturated fatty acid, PE phosphatidylethanolamine, ROS reactive oxygen species, RSL3 Ras-selective lethal 3, STEAP3 six-transmembrane epithelial antigen of prostate 3 metalloreductase, SLC7A11 solute carrier family 7 member 11, TF transferrin, TFR1 transferrin receptor 1, VDAC2/3 voltage-dependent anion channel 2/3.

Iron is one of the most important trace elements in the body. Normal physiological processes can be affected by abnormal distribution and content of iron in the body [18]. There are four main aspects to iron metabolism: absorption, storage, utilization, and excretion [22]. Iron ingested from food is absorbed by the epithelial cells of the duodenum, reduced to Fe2^+^ through iron reductase in the intestinal epithelial cells, and transported to the cells by divalent metal transporters [23]. Circulating Fe3^+^ enters cells via the TF/TFR-1 transport system and is reduced to Fe2^+^ by the metal reductase STEAP3. The reduced Fe2^+^ is transferred to the cytoplasm via DMT1 and is involved in a variety of subsequent physiological and biochemical processes, including DNA biosynthesis, oxygen transport, and regulation of metabolic pathways [24]. Fe2^+^ in the cytoplasm initially forms various iron-binding complexes. When the iron-binding complexes reach saturation, the excess Fe2^+^ accumulates in an unstable iron pool. The Fe2^+^ in the liable iron pool participates in the Fenton reaction to generate ROS, mainly hydroxyl radicals. This leads to membrane lipid peroxidation and ultimately ferroptosis [25]. Heat shock protein beta-1 (HSPB1) inhibits TFR-1 expression and reduces intracellular iron concentration [26]. Ferritin consists of two subunits, FTL and FTH1. It is the primary protein that stores iron ions [24]. The Nuclear receptor coactivator 4 (NCOA4) is an important molecule that mediates ferroptosis, and silencing NCOA4 decreases the level of liable iron pools and inhibits ferroptosis [14, 27]. However, by enhancing ferritinophagy through inhibition of cytosolic glutamate oxaloacetate transaminase 1 (GOT1), the level of labile iron pools increases, ultimately leading to ferroptosis [28] (Fig. 1).

### Lipid metabolism

PUFAs are significant components of the phospholipid bilayer and are crucial in regulating cell membrane fluidity. Nonetheless, PUFAs (such as arachidonic acid and epinephrine) are vulnerable to the Fenton reaction, which produces excessive peroxides that impair the phospholipid bilayer structure, thereby compromising cell membrane function [29–31]. Acyl-CoA synthase long-chain member 4 (ACSL4) and lysophosphatidylcholine acyltransferase 3 (LPCAT3) enzymes are required for the biosynthesis and remodeling of polyunsaturated fatty acids [31]. Phosphatidylethanolamine (PE) is a glycerophospholipid present in cellular membranes. In mitochondria, PE makes up 40% and in other organelles, membrane accounts for about 15%–25% [32, 33]. PUFAs react with coenzyme A to produce acyl-CoA through the catalytic action of ACSL4 [34–37]. Subsequently, LPCAT3 facilitates the conversion of acyl-CoA to membrane phosphatidylethanolamine through esterification, resulting in the production of PUFA-PE [36, 38, 39]. Finally, the oxidation of polyunsaturated fatty acids (PUFA-PE) by LOX results in cellular ferroptosis [37] (Fig. 1).

### Amino acid metabolism

Glutathione (GSH) is a tripeptide consisting of glycine, cysteine, and glutamic acid-containing Y-amido-hexapeptide and sulfhydryl groups, which is an important antioxidant [40, 41]. GSH functions intracellularly as a vital substrate for GPX4 [42]. The cell membrane features a class of heterodimers known as System Xc-, which is comprised of Solute Carrier Family 7 Member 11 (SLC7A11) and Solute Carrier Family 3 Member 2 (SLC3A2) [43]. The System Xc- enables the transfer of extracellular cystine to intracellular glutamate in a 1:1 proportion, which is succeeded by the production of GSH from intracellular cystine, aided by the actions of glutamate-cysteine ligase (GCL) and glutathione (GSS) [42, 44]. Cystine uptake in the majority of mammalian cells is mediated by SLC7A11, which is then followed by a reduction reaction that depletes nicotinamide adenine dinucleotide phosphate (NADPH) to produce cysteine [41]. Cysteine is a member of the group of amino acids containing sulfur. Additionally, cysteine can be converted from methionine through the transsulfuration pathway [45]. GPX4 and System Xc- play crucial roles in the amino acid metabolism of ferroptosis. Furthermore, GPX4 coenzyme GSH works to convert phospholipid peroxides to phospholipids, thereby shielding cells from ferroptosis [45]. It has been demonstrated that ROS primarily stem from the electron transport chain (ETC) complex within mitochondria. Inhibition of the ETC results in diminished accumulation of oxides, thereby hindering ferroptosis [46]. ROS production can impact the regulatory function of ferroptosis. Additionally, the regulation of ferroptosis can be influenced by mitochondrial tricarboxylic acid (TCA), and the inhibition of the TCA cycle in mitochondria can block the voltage-dependent channel 2/3 (VDAC2/3) to safeguard cells from ferroptosis [47]. In conclusion, the regulation of ferroptosis is closely linked to amino acid metabolism (Fig. 1).

## Physiological functions of ferroptosis

Ferroptosis was initially introduced in 2012 as a form of cell death reliant on iron. Current research has found a close link between ferroptosis and a range of physiological processes. Biological processes connected to ferroptosis were primarily advanced through exploring markers of ferroptosis [48]. In tumor suppression and immune functions, many anti-oncogenes have been discovered to exert fractional tumor-suppression function by inducing ferroptosis. For instance, the tumor-suppressor gene p53, which is involved in tumor development, inhibits the expression of SLC7A11 [9]. Moreover, MLL4 deletion results in a significant suppression of ferroptosis in cutaneous squamous cell carcinoma [49]. Likewise, the tumor suppressive effects of BAP1 and NFS1 rely on the regulation of ferroptosis [50]. PUFA (Polyunsaturated fatty acid)-rich diets increase the production of PUFA-containing phospholipids (PLs), which promote tumor ferroptosis [30]. CD8^+^ T cells facilitate tumor cell ferroptosis by releasing IFNγ and inhibiting the expression of SLC7A11 [51]. CD8^+^ T cells also release AA (arachidonic acid), promoting tumor cell ferroptosis [48]. High-cholesterol foods enhance the expression of CD36 on the surface of CD8^+^ T cells, leading to the uptake of PUFAs in CD8^+^ T cells [52]. Selenium supplementation has been found to promote antiviral immunity. Selenium-enriched diets improved the expression of the selenoprotein GPX4, which inhibited ferroptosis in CD4^+^ TFH cells, promoting memory B cells and persistent viral immunity [53]. Ferroptosis markers increased with age in the brain tissues of rats and mice. Similarly, with increasing age, iron gradually increased, and GSH gradually decreased in nematodes, ultimately leading to ferroptosis. Ferroptosis was identified in nucleated erythrocytes before enucleation and maturation, suggesting its role in both the normal erythropoiesis process and the aging of various organs [48].

## Ferroptosis and inflammation

**Fig. 2:**
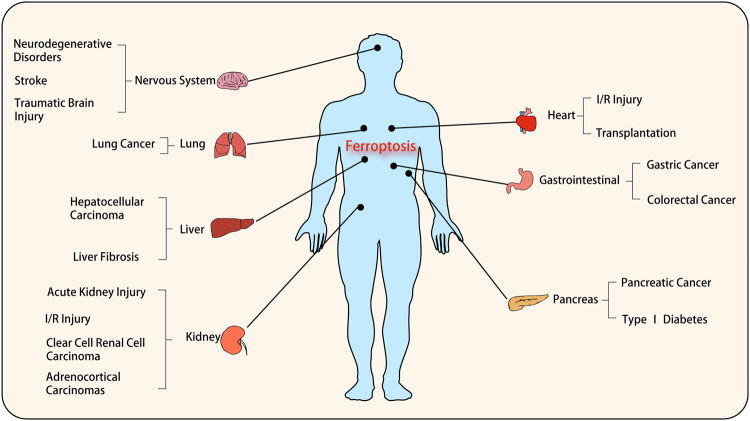
Ferroptosis has played important roles in multiple system diseases.

Abnormal activation of ferroptosis in many organs is involved in the development of many tumors or pathological processes (Fig. 2).

Inflammation is a crucial and innate physiological activity in the body. While a moderate inflammatory response benefits the organism, an excessive response can result in harm. The body initiates inflammation in response to exposure to various damaging factors. Clinically, inflammation often presents with redness, heat, swelling, and dysfunction. Inflammation-inducing agents related to lipid peroxidation and AA metabolism manifest during ferroptosis. AA is a Polyunsaturated fatty acid primarily present as PLs in cell membranes. When cell membranes are exposed to various stimuli, vast amounts of AA are released from PLs by phospholipase A2 (PLA2) and phospholipase C (PLC) [54–56], which is then metabolized into biologically active inflammatory mediators like eicosanoids prostaglandin (PG), interleukin (IL), tumor necrosis factor (TNF), and leukotriene (LT). These mediators can contribute to the inflammatory cascade. AA metabolism is involved in at least three metabolic pathways, namely the COX pathway, the LOX pathway, and the cytochrome p45 (CYP450) pathway [57]. The COX and LOX pathways are the primary routes for metabolizing AA into inflammatory mediators and breaking it down into leukotrienes, prostaglandins, and some peroxides [58]. COX enzymes facilitate the conversion of AA into prostaglandins (PGs). Two isoforms of COX exist, namely COX1 and COX2. COX1 is a beneficial enzyme widely present in various cell types, while COX2 is an inducible enzyme encoded by the PTGS2 gene. COX2 plays a vital role in activating macrophages or other inflammatory cells [59]. COX2 expression is minimal in normal physiological conditions but abundant in inflammatory cells. This phenomenon can lead to the transformation of inflammatory regions into pre-tumor microenvironments [60, 61]. COXs convert arachidonic acid to prostaglandin G2 (PGG2) and prostaglandin H2 (PGH2), which generate a variety of biologically active PGs, including PGE2, prostaglandin D2 (PGD2), and prostaglandin I2 (PGI2), through the action of various isoenzymes. PGH2 is highly unstable and has a half-life of approximately 30 seconds. In the presence of thromboxane synthetase, thromboxane A2 (TXA2) may also be produced [62, 63]. Increased expression and release of PTGS2 in the prostate is associated with ferroptosis, which promotes arachidonic acid metabolism and the release of inflammatory factors through the upregulation of PTGS2 [11]. LOX is not only involved in oxidative lipidation but is also an important signal for ferroptosis, which also converts AA to inflammatory mediators such as leukotrienes (LTs) and lipoxygenase (LXs) [64]. The CYP450 pathway has the ability to metabolize arachidonic acid into epoxyeicosatrienoic acid (EET) and hydroxyeicosatetraenoic acid (HETE) [65]. It is hypothesized that ferroptosis may be closely associated with arachidonic acid metabolism and the inflammatory response. However, the precise mechanism of action remains unclear.

## Ferroptosis and cancer

### BAP1-mediated regulation of ferroptosis

The BAP1 gene, associated with the suppression of tumors, encodes a nuclear deubiquitinating (DUB) enzyme that forms the polycomb repressive deubiquitinase (PR-DUB) complex. This complex epigenetically regulates gene expression by decreasing histone 2 A ubiquitination (H2Aub) on chromatin in nucleosomes [66–71]. The tumor-suppressor activity of BAP1 is mediated, in part, by the repression of SLC7A11 expression through deubiquitinating of H2A on the SLC7A11 promote. SLC7A11 facilitates the cellular uptake of cystine, which is the primary precursor for glutathione (GSH) biosynthesis. GSH is a crucial molecule for cellular resistance to oxidative stress [72]. Inhibition of SLC7A11 by the erastin increases lipid peroxidation, leading to ferroptosis [73]. The study found that overexpression of WT BAP1 in UMRC6 cells hindered the cellular uptake of cystine, resulting in reduced GSH levels and increased erastin-induced lipid peroxidation [50]. BAP1 suppresses the expression of SLC7A11 to inhibit cystine uptake, leading to lipid peroxidation and ferroptosis. SLC7A11, the cystine transporter protein, was identified as a critical BAP1 target gene in human cancers. Moreover, BAP1 mutants associated with cancer were found to lose their ability to repress SLC7A11 and to promote ferroptosis. In vivo experiments indicate that restoring BAP1 expression in BAP1-deficient cells suppresses the growth of xenografted tumors. Human tumor-associated mutations in BAP1 do not inhibit the expression of SLC7A11 and do not suppress tumors via promoting ferroptosis [50].

### P53-mediated regulation of ferroptosis

As a crucial suppressor gene of tumors, p53 takes part in numerous biological processes, including cell cycle inhibition, aging, and apoptosis. Almost half of the human tumor tissues display mutations or inactivation of the p53 gene. In addition, p53 may have antitumor effects by regulating intracellular ferroptosis. Specifically, under oxidative stress, p53 can either promote or inhibit ferroptosis [74]. Its pro-death functions include inhibiting SLC7A11 expression and promoting SAT1 expression [75]. Additionally, acetylation modification of the P53 DNA-binding region can regulate SLC7A11 expression, thereby promoting ferroptosis in certain cancer cells [9]. Notably, the acetylation-deficient mutant P533KR inhibits SLC7A11 expression and increases cell sensitivity to ferroptosis [76]. However, the mutant p534KR did not down-regulate SLC7A11 expression [76]. Additionally, p53 regulates ferroptosis by targeting SAT1 (spermine/spermine n1-acetyltransferase 1) [77, 78]. Silencing SAT1 significantly abrogates p53-mediated ferroptosis, while upregulation of SAT1 expression sensitizes cells to ferroptosis upon hypoxic conditions. Mechanistically, SAT1 did not affect the expression or activity of SLC7A11 and GPX4. Furthermore, the P53-SAT1-ALOX15 pathway regulates ferroptosis [77]. Another important aim in regulating ferroptosis during glutamine metabolism is the targeting of Glutaminase 2 (GLS2), a crucial mitochondrial glutaminase that has recently been studied and recognized as a fresh transcriptional objective of p53 [79]. Expression in tumor cells is linked to mitochondrial oxidative stress and ATP synthesis regulated by p53. In HepG2, LN-2024, and HCT116 cells, the expression of GLS2 facilitates the generation of glutathione in tumor cells, thus enhancing cellular antioxidant function [80]. Therefore, GLS2 may be a negative regulatory protein for ferroptosis.

**Fig. 3:**
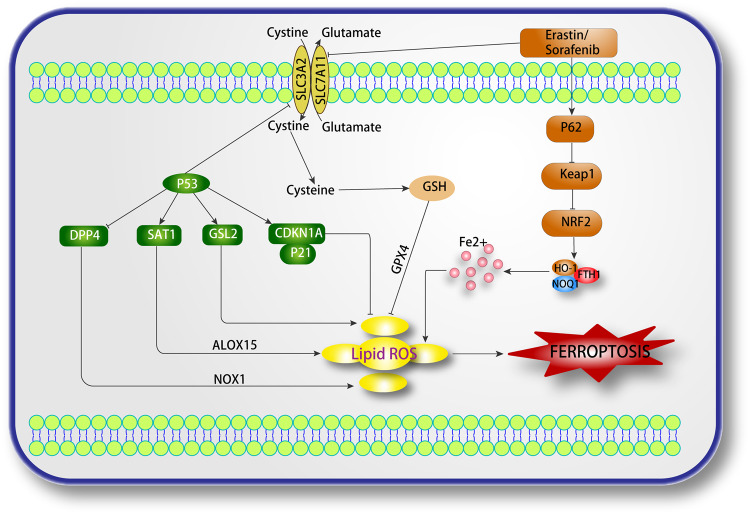
Signaling pathways regulating ferroptosis in cancer. ALOX arachidonate-15- lipoxygenase, CDKN1A cyclin-dependent kinase inhibitor 1A, DPP4 dipeptidyl-peptidase-4, GLS2 glutaminase 2, GSH glutathione, GPX4 glutathione peroxidase 4, NOX1 NADPH oxidase 1, ROS reactive oxygen species, SAT1 spermidine/spermine N1-acetyltransferase 1, SLC7A11 solute carrier family 7 member 11, HO-1 heme oxygenase 1, FTH1 ferritin heavy chain 1, NRF2 nuclear factor erythroid 2-related factor 2 NRF2.

On the other hand, the pro-survival functions of p53 in ferroptosis include inhibiting dipeptidyl-peptidase 4 (DPP4) activity and promoting the promotion of CDKN1A/p21 expression. Research has shown that p53 has a pro-survival function in ferroptosis inhibition by blocking DPP4 activity. Knockout, knockdown, or pharmacologically inhibiting p53 boosted the anticancer efficacy of type I ferroptosis inducers (erastin and sulfasalazine). However, it had no significant effect on tumor cell killing by type II ferroptosis inducers (RSL3 and FIN56). DPP4 inhibitors completely blocked erastin-induced ferroptosis in CRC cells. When p53 is absent, DPP4 triggers membrane-associated DPP4-mediated lipid peroxidation through binding to NOX1 (NADPH oxidase 1) and forming the NOX1-DPP4 complex, leading to ferroptosis in CRC cells [81]. NADPH oxidases (NOX) are a group of proteins that transfer electrons into the cell and reduce oxygen to superoxide anion. NOX1 facilitates ROS production via NADPH, contributing to erastin-induced ferroptosis [1]. CDKN1A can achieve anti-oxidative stress by inhibiting apoptosis. CDKN1A/p21 plays an important role in p53-mediated DNA damage-induced cell cycle arrest [82]. It has been reported that the regulation of CDKN1A by p53 delays ferroptosis occurrence in tumor cells when Cys (cysteine) is absent. The activation of CDKN1A/p21-mediated GSH metabolism by p53 can inhibit cellular ferroptosis (Fig. 3).

### NRF2-mediated regulation of ferroptosis

NRF2, encoded by the NFE2L2 gene, is precisely regulated by the E3-ubiquitin ligase system [83–85]. It can specifically target and regulate a diverse range of proteins that are closely linked to the ferroptosis cascade. It should however be noted that NRF2 activation can potentially trigger tumor progression and resistance to therapy [86]. While NRF2 inducers are reported to have a protective effect on normal cells from carcinogens, NRF2 inhibitors are useful in reducing the resistance to ferroptosis in patients with tumors [87]. Some HNC cells were found to evade ferroptosis caused by inhibition of GPX4, and HNC cells insusceptible to RSL3 or ML162 were resistant to ferroptosis induced by GPX4 inhibitors. However, the resistance was reduced by inhibition of Keap1 or upregulation of the NRF2 gene [88]. Additionally, the research discovered that the expression of NRF2 was considerably enhanced in HNC cells following the usage of artesunate. Furthermore, the deletion of NRF2 resulted in decreased resistance to ferroptosis caused by artesunate due to GSH deficiency and heightened ROS levels both in vivo and in vitro [89]. It has been reported that NRF2 is significantly expressed in various malignancies and is closely linked to the malignant phenotype and patient prognosis [90]. Additionally, it has been uncovered that NRF2 improves the chemosensitivity of tumor cells to sorafenib-induced ferroptosis [90]. Sun et al. [91] demonstrated that quiescin sulfhydryl oxidase 1 (QSOX1) inhibited NRF2 activation during sorafenib-induced ferroptosis in HCC cells. A different research team also indicated that erastin, sorafenib, and buthionine sulfonimine, which are ferroptosis inducers, augmented NRF2 expression in HCC cells [92]. Conversely, the downregulation of NRF2 by FTH (ferritin heavy chain), NQO1 (NAD(P)H quinone oxidoreductase-1), and HO-1 (heme oxygenase1) amplified the sensitivity to ferroptosis in HCC [92]. Additionally, Glutathione S-transferase zeta 1 (GSTZ1) [93] and Sigma-1 receptor (SIR) [94] improved the sensitivity of HCC cells to sorafenib-induced ferroptosis by down-regulating NRF2. High NRF2 expression stimulated GBM cell proliferation and facilitated the oncogenic transformation. Furthermore, the activation of NRF2 was observed to confer resistance to erastin- and RSL3-induced ferroptosis in GBM cells [95] (Fig. 3).

### EMT-mediated regulation of ferroptosis

Epithelial-mesenchymal transition (EMT) refers to the process whereby epithelial cells lose their polarity and intercellular adhesion while acquiring mesenchymal properties associated with invasive and migratory capabilities [96]. Research has demonstrated that transcription factors, including SNAI1, TWIST1, and ZEB1, can advance EMT and promote drug resistance in tumor cells [97]. These findings might be potential therapeutic targets in oncology. Furthermore, EMT signaling promotes ferroptosis. 2,2’-Di-pyridylketone hydrazone dithiocarbamate s-butyric acid (DpdtbA), an iron chelator, exhibited potent antitumor effects in gastric and esophageal cancer cells. DpdtbA can also inhibit EMT by activating the P53 and PHD2/HIF1α pathways [98]. Furthermore, the use of an iron chelator reduced cigarette smoke exposure (CSE)-induced EMT mitochondrial malfunction and cell death in lung epithelial cells. Iron chelators were identified as inhibitors of cell death induction after RSL3 treatment [99]. In addition, The expression of Bach1 was increased in glioma cells and was strongly associated with EMT. Notably, the development of ferroptosis is inhibited by overexpression of Bach1 in glioma cells [100]. The TGFβ family comprises three TGFβs, two activins, numerous bone morphogenetic protein (BMP) homologs, and various homodimers and heterodimers of ligands. Intracellular lipid peroxidation is induced by TGF-β1, which promotes EMT in melanoma cells and consequently strengthens cellular ferroptosis [101]. In addition, e-cadherin-mediated cell-to-cell contact activates the Hippo pathway, ultimately reducing a process known as ferroptosis. This, in turn, inhibits the activity of YAP, a protein that co-regulates transcription associated with ferroptosis. YAP is activated after the initiation of EMT, therefore increasing cells’ vulnerability to ferroptosis [102]. It has also been found that incorporating histone deacetylase (HDAC) inhibitors can promote EMT in SW13 cells, further increasing their sensitivity to ferroptosis [103].

### HIF-mediated regulation of ferroptosis

Hypoxia is a crucial factor in promoting tumor growth and treatment resistance [104]. HIF (Hypoxia‑inducible factor), an essential regulator of hypoxia, is made up of an oxygen-unstable subunit, including HIF1α, EPAS1 (HIF2α), and HIF3α, and an expression-regulated subunit, ARNT [105]. Under normal oxygen levels, HIF1α and HIF2α are hydroxylated by members of the EGLN family. Subsequently, HIF1α and HIF2α bind to E3 ubiquitination ligase, VHL, and are eventually degraded through the proteasome pathway. Under hypoxia conditions, the inactivation of hydroxylase results in the intracellular accumulation of HIF-1α, HIF2α, and ARNT to form heterodimers, which regulate cellular adaptation to hypoxia and survival. HIF-1α and HIF-2α are aberrantly expressed in tumors and intimately linked to patient prognosis [106]. In renal clear cell carcinoma, HIF is a key driver of ferroptosis. Further, the inclusion of HIF while inhibiting GPX4 mitigates GPX4-mediated ferroptosis. HIF-1α and HIF-2α have been identified as regulators of ferroptosis in renal clear cell carcinoma cells [107]. Inducing the expression of HIF-1α through increasing the expression of fatty acid-binding proteins 3 and 7 under hypoxic conditions inhibits ferroptosis [108]. Solute carrier family 1 member 1 (SLC1A1) was found to be a membrane glutamate transporter that functions to transport extracellular glutamate into the cell, driving SLC7A11-mediated cystine uptake [109]. Similarly, in gastric cancer cells, HIF-1α inhibits ferroptosis by up-regulating the level of SLC7A11 [107]. By contrast, HIF-2α activated in RCC-derived cells enhanced polyunsaturated lipids and caused lipid peroxidation through hypoxia-inducible, lipid droplet-associated protein (HILPDA) activation [8]. Moreover, scholarly articles report that HIF-2α activation in colorectal cancer notably increases gene expression relating to lipid and iron metabolism, promoting the cell prone to ferroptosis [8].

## Targeting ferroptosis in cancer treatment

Tumor resistance to chemical drugs poses a significant challenge to oncology treatment. Ferroptosis, a distinct form of cell death, plays a crucial role in inhibiting tumor growth and has opened new avenues for treating chemotherapy-resistant tumors. A mounting body of research has highlighted ferroptosis as a possible target for overcoming chemotherapy resistance [6, 7, 110]. Therefore, ferroptosis inducers have a pivotal role in treating tumor resistance.

## Ferroptosis and acquired drug resistance in cancers

Inducing “ferroptosis” is a viable method of combating drug-resistant tumor cells. Recent research has demonstrated that YAP/TAZ potentially facilitates the resistance of hepatocellular carcinoma cells to sorafenib-induced ferroptosis through the co-regulation of SLC7A11 expression with ATF4. Thus, YAP/TAZ could constitute a crucial molecular mechanism regulating ferroptosis in hepatocellular carcinoma cells while also promoting the emergence of sorafenib resistance [111]. One of the key molecular mechanisms that regulate ferroptosis in hepatocellular carcinoma cells and promote sorafenib resistance. In particular, NRF2 exhibits the capacity to regulate the expression of multiple target genes, including GPX4 and SLC7A11, within the SLC7A11-GPX4-GSH pathway. Consequently, this can influence the susceptibility of tumor cells to drug treatment [86, 112, 113]. Furthermore, ZEB1 expression was significantly upregulated in stromal cells resistant to chemotherapy but appeared to be more susceptible to ferroptosis induced by statin treatment or GPX4 inhibition [7]. Additionally, ferroptosis was found to be effective in treating drug-resistant neuroblastomas. Ferritin A-induced ferroptosis effectively eradicated high-risk neuroblastoma cells and inhibited their growth [114].

## Ferroptosis inducers for cancer therapy

### Targeting system Xc-

System Xc- inhibitors can impact the uptake of Cys in cells, thereby affecting protein folding regulation. The incomplete protein folding accumulates and creates cellular stress, which ultimately leads to cellular ferroptosis. By inhibiting System Xc- directly, erastin can reduce GSH levels [115]. In RAS-bearing tumor cells, RAF/MEK/ERK is a vital pathway for erastin-induced ferroptosis. The mitochondrial voltage-dependent anion channel (VDAC) is a targeted molecule of erastin. Knockdown of VDAC2/3 leads to resistance to erastin. Erastin induces ferroptosis in tumor cells and enhances the therapeutic ability of conventional antitumor drugs such as doxorubicin and cisplatin [116, 117]. However, erastin is challenging to dissolve in water and metabolically unstable in vivo. Piperazine erastin (PE) has superior water solubility and stability compared to erastin [11]. Furthermore, significant enhancements have been made to the water solubility and anticancer performance of Ketone erastin [118].

Sulfasalazine (SAS) is a prevalent anti-inflammatory drug in clinical practice. The FDA categorizes SAS as a primary medication for treating rheumatoid arthritis [119]. Additionally, it has been discovered that SAS triggers intracellular ferroptosis by hindering System Xc-. However, SAS is significantly less potent than erastin. Studies have shown that SAS can induce ferroptosis in various types of tumor cells, including HT-1080, Calu-1, and 143 B. Additionally, it is beneficial as a combination therapy in improving the outcome of gliomas [115].

Sorafenib is a multikinase inhibitor that demonstrates effective treatment in advanced cancers such as renal cell carcinoma, hepatocellular carcinoma, and thyroid cancer [115]. Although, there is evidence that some tumor cells have developed resistance to sorafenib treatment. Sorafenib has the capability to inhibit ferroptosis in HCC through the activation of NRF2 and Rb, among others [120]. The high affinity of the NRF2 transcription factor MT-1G in cells is associated with this phenomenon [121]. Blocking the activation of the MT-1G signaling pathway using sorafenib may decrease resistance to sorafenib treatment and increase efficacy [122]. Ferroptosis can be triggered in some tumor cells via blockade of System Xc- mediated cystine uptake, but transcriptional activation of the transsulfuration pathway in other tumor cells allows them to convert from methionine to cysteine. Consequently, System Xc- inhibitors cannot induce cellular ferroptosis in these cells [123].

### Induced degradation of GPX4

In addition, the induction of ferroptosis can be achieved through the regulation of GPX4 levels, a key regulatory protein for ferroptosis, which has a crucial protective role against the process in organisms. GPX4 utilizes GSH as a substrate to reduce ROS to the corresponding fatty alcohols and prevent ROS accumulation [124]. GSH, an antioxidant, has been long recognized as a weak spot of cancer [125, 126]. Direct GPX4 activity targeting has the potential to trigger ferroptosis in cells [27]. Studies have revealed that antitumor medications heavily rely on GPX4. In vitro, administration of GPX4 inhibitors eliminates cancer cells and hinders their reappearance in vivo. The molecule RSL3 directly curbs GPX4 expression, impeding ferroptosis [6, 127]. RSL3 interacts with enzymes involving nucleophilic interactions, such as cysteine, serine, and selenocysteine, and modifies GPX4, rendering the enzyme inactive [123].

FIN56, a ferroptosis inducer derived from CIL56, demonstrates greater selectivity for ferroptosis in comparison to its parent compound. It catalyzes GPX4 degradation through ACC mediation. In addition, FIN56 induced coenzyme Q10 (CoQ10) depletion through interaction with squalene synthase (SQS), thereby increasing the sensitivity of cells to ferroptosis [128]. Furthermore, withaferin A promotes neuroblastoma (NB) cell death by increasing LIP levels through Hmox1, in addition to inactivating GPX4 [114]. BAY 87-2243, a known inhibitor of IκBa, upregulates Hmox1 in a nuclear factor manner independent of kb, thus promoting iron accumulation and inducing ferroptosis in tumor cells [129]. Additionally, FINO2, an endogenous peroxidase 1, 2-dioxygenase, induces ferroptosis in tumor cells by oxidizing iron and inactivating GPX4 [130].

### Nanoparticle inducers

In recent years, Nano ferroptosis inducers have attracted great attention for cancer therapy [131, 132]. Nanoscale ferroptosis inducers can be loaded onto nanocarriers to enhance solubility and biocompatibility [118]. Furthermore, nanomaterials have the potential to disturb the metabolic balance of an organism by interfering with intracellular biochemical processes, which can result in intracellular ferroptosis [123]. The initial discovery of a nanoparticle ferroptosis inducer, αMSH-PEG-C’ dots, was documented in 2016 [133]. As observed in cancer cells, injecting this inducer caused an escalation in intracellular iron levels, accompanied by ROS production and GSH depletion, ultimately triggering ferroptosis [134]. An alternative method involves manufacturing iron-containing nanoparticles capable of delivering and releasing iron to cancerous cells [135, 136]. Liu et al. devised a technique that engrosses Fe3^+^ and tannic acid (TA) onto the surface of SRF, resulting in SRF@Fe^IIII^TA (SFT). Furthermore, SFT experiences damage to its nuclear-corona nanostructure when subjected to a lysosomal micro-acidic environment, eliciting ferroptosis via the inhibitory properties of SRF on GPX4. Additionally, tannic acid exhibits a potent acidic reducing ability, reducing Fe3^+^ to Fe2^+^ and generating reactive oxygen species, thereby promoting ferroptosis [137]. Shen et al. attempted to increase the concentration of each reactant (Fe3^+^, Fe2^+^, H_2_O_2_) under different conditions in the system to accelerate ROS generation [138]. Aside from regulating the metabolism of iron and reactive oxygen species, nanomaterials can also trigger iron-dependent cell death by controlling GSH levels [139]. In addition to the aforementioned, Wang et al. have produced an inducer of iron death using arginine-rich manganese silicate nanobubbles, specifically arginine-capped manganese silicate nanobubbles (AMSNs), which possess a notable ability to scavenge GSH [140]. In conclusion, the use of ferroptosis inducers is of paramount importance in tumor therapy.

## Conclusions

Ferroptosis is a newly discovered type of cell demise literature that plays a significant role in numerous diseases. This review discusses the molecular mechanisms of ferroptosis in cancer and proposes new targets for clinical therapy. In addition, the presence of inflammatory mediators associated with arachidonic acid metabolism during ferroptosis and the potential positive impact of ferroptosis on inflammation, which may aid in the treatment of related inflammatory diseases. However, the relationship between ferroptosis and apoptosis requires more exploration. Further exploration and investigations into these issues are necessary for scientists to gain a better understanding of the molecular mechanics behind ferroptosis. These could help provide new concepts and methodologies for clinical treatment.
